# Lemierre’s Syndrome Caused by Klebsiella pneumoniae: A Case Report and Literature Review

**DOI:** 10.7759/cureus.44434

**Published:** 2023-08-31

**Authors:** Tao an Chen, Ya Ting Chuang, Hua Yu Lin, Cheng Hsien Chen

**Affiliations:** 1 Division of Respiratory Therapy, Department of Chest Medicine, Show Chwan Memorial Hospital, Changhua, TWN; 2 Surgical Intensive Care Unit, Department of Nursing, Show Chwan Memorial Hospital, Changhua, TWN; 3 Department of Surgery, Show Chwan Memorial Hospital, Changhua, TWN

**Keywords:** neck swelling, septic embolism, diabetes mellitus, klebsiella pneumonia, lemierre's syndrome

## Abstract

Lemierre's syndrome is a rare condition that involves anaerobic sepsis following pharyngitis and is characterized by a high mortality rate. It often manifests as a septic embolism within the internal jugular vein due to oropharyngeal infections, leading to vein wall inflammation. Despite modern antibiotics, Lemierre's syndrome remains underdiagnosed and poses a significant threat. We report the case of a 43-year-old man who has alcoholic liver cirrhosis and diabetes mellitus. Symptoms included chest pain, back pain, and neck swelling, with *Klebsiella pneumoniae* leading to the diagnosis of *K. pneumoniae-*associated Lemierre’s syndrome. Furthermore, *K. pneumoniae-*associated Lemierre's syndrome is linked to diabetes mellitus and the elderly population. Notably, it showed a tendency for distant metastases, particularly in the lungs and brain. Additionally, central nervous system and renal involvement were observed in a smaller subset of cases.

## Introduction

Lemierre's syndrome was first described in a series of cases in 1936 [1]. It is commonly presented as unilateral internal jugular vein septic embolism, mostly caused by oropharyngeal infections, which subsequently lead to inflammation within the vein wall [2-3]. Pulmonary complications were frequently observed [4-5]. As of today, it remains a rare syndrome with an estimated worldwide incidence of three to six cases per million. It occurs especially in otherwise healthy young adults and is more likely to occur in men than in women [1,5]. The most common pathogen is Fusobacterium species (e.g., *Fusobacterium necrophorum*). *Klebsiella pneumoniae* is a relatively rare pathogen [6]. We report a case of Lemierre's syndrome caused by *K. pneumoniae* and conduct a literature review. We aim to identify the clinical characteristics of such patients and compile relevant medical management recommendations to share with peers through this case report and literature review.

## Case presentation

A 43-year-old man has a history of alcoholic liver cirrhosis and diabetes mellitus (DM). He visited the emergency room due to chest pain, back pain, and swelling on the right side of his neck that had persisted for five days. Upon examination, his vital signs were stable and he had no fever. The family representative reports that there is no history of allergies to food, medication, or blood transfusions. The individual has a history of smoking and alcohol consumption but has been abstinent for three years. There is no travel history, special occupational history, specific disease contact history, or association with any clusters. In the subsequent laboratory tests, we observed elevated levels of blood urea nitrogen, creatinine, C-reactive protein, lactic acid N-terminal pro-B-type natriuretic peptide, neutrophil segments, and procalcitonin. In the complete blood cell count, we observed elevated neutrophil segment levels and a decreased platelet count (Table 1). However, the subsequent electrocardiogram examination revealed normal results.

**Table 1:** Laboratory results at the time of admission.

Test	Result (on admission)	Reference range
Blood urea nitrogen	31 mg/dL	6–20 mg/dL
Creatinine	1.37 mg/dL	0.7–1.2 mg/dL
Creatine phospho-kinase	51 U/L	39–308 U/L
N terminal pro-B-type natriuretic peptide	815 pg/mL	<125 pg/mL
Creatine kinase MB	1.11 ng/mL	<6.22 ng/mL
Troponin T	7.7 ng/L	<14 ng/L
D-Dimer	13.48 µg/mL	<0.5 µg/mL
Lactic acid	74 mg/dL	4.5–19.8 mg/dL
C-reactive protein	16.6 mg/dL	<0.5 mg/dL
Procalcitonin	8.6 ng/mL	<0.5 ng/mL
White blood cell	4980/µL	4500–10,000 /µL
Neutrophil segment	92%	40–74%
Platelet	2.5 × 10^4^/µL	13–40 × 10^4^/µL

**Figure 1:**
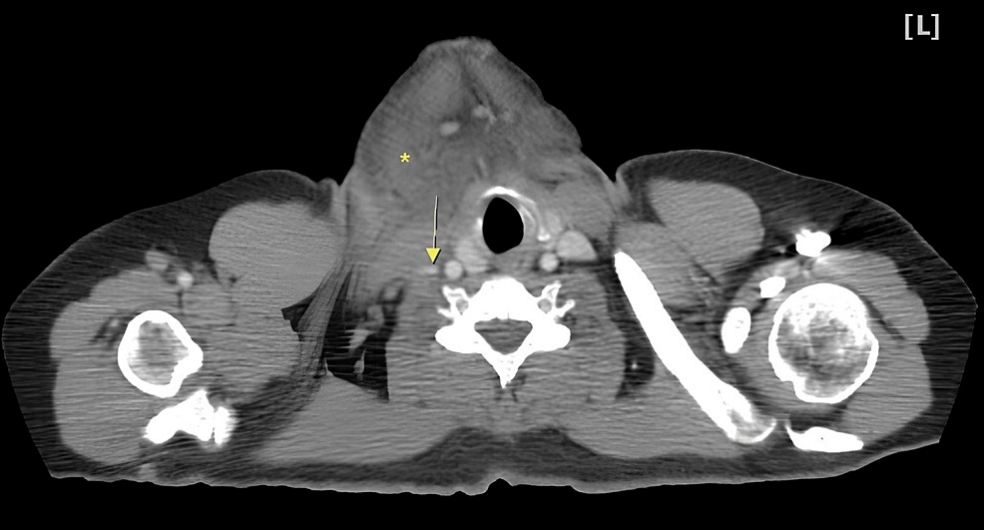
The computed tomography of the neck showed right neck swelling and reduced compressive blood flow, thrombophlebitis and a small amount of thrombus found in the right internal jugular vein.

**Figure 2:**
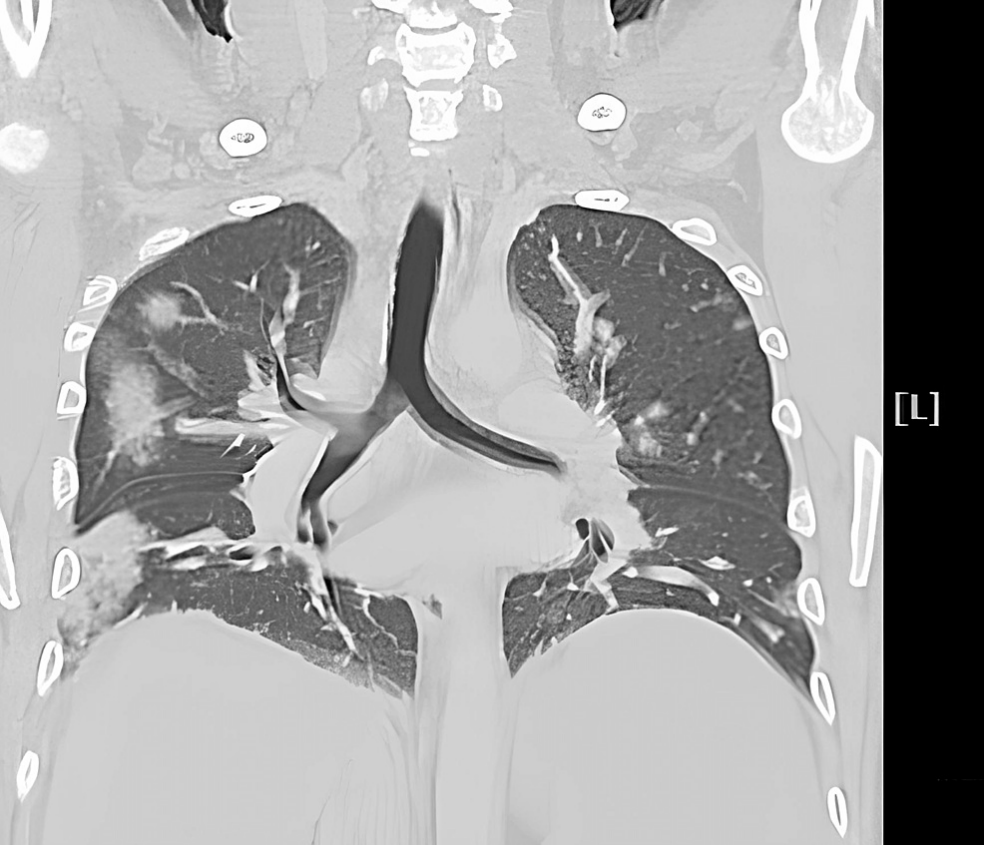
The computed tomography of the thorax showed multiple nodular and patchy infiltrates at right lung, and septic pulmonary embolism could be identified.

The computed tomography (CT) scan of the neck showed right neck swelling (Figure 1, asterisk) and the right internal jugular vein reduced compressive blood flow, thrombophlebitis, and a small amount of thrombus (Figure 1, arrow). The CT of the thorax displayed multiple nodular and patchy infiltrates in the right lung, and a septic pulmonary embolism could be identified (Figure 2). In terms of antibiotic therapy, we give meropenem at a dose of 1 g intravenously every eight hours to cover the potential infections after admission. The patient was then transferred to the intensive care unit. We detected *K. pneumoniae* in blood and sputum cultures. Additionally, approximately eight hours after admission to the intensive care unit, the patient began to experience a continuous fever ranging from 38 °C to 39 °C. As a result, the patient was diagnosed with Lemierre's syndrome. Unfortunately, the patient developed type 2 respiratory failure and was intubated. Regrettably, the patient passed away the following day.

## Discussion

In 1936, Andre Lemierre reported a series of 20 cases of post-pharyngitis anaerobic sepsis with a high mortality rate [1]. The syndrome commonly presents as unilateral internal jugular vein septic embolism, primarily caused by oropharyngeal infections, subsequently leading to inflammation of the vein wall [2-3]. As of today, it remains a rare syndrome with an estimated worldwide incidence of three to six cases per million. It predominantly affects otherwise healthy young adults, with a higher prevalence in men than women [1,5]. Despite the availability of appropriate treatments, the mortality rate remains as high as 20% [7]. In the context of widespread antibiotic use in the modern era, Lemierre's syndrome is often overlooked in diagnoses, earning it the moniker of the "forgotten disease" [6-7].

Lemierre's syndrome can be caused by various pathogens, among which fusobacterial species (e.g., *F. necrophorum*) are the most common [6]. *K. pneumoniae* is also one of the possible pathogens. Recent literature indicates that *K. pneumoniae* causes about 2.3-2.5% of cases [6-7].

As cases related to *K. pneumoniae* continue to be reported, we performed a PubMed literature search for English-language articles. We were using the search terms "Klebsiella pneumoniae" and "Lemierre’s syndrome" and published from inception to August 2023. Consequently, we found 12 cases of *K. pneumoniae*-associated Lemierre’s syndrome (KLS) and summarized the 13 cases, including the case being reported by us (Table 2).

**Table 2:** Summary of cases of Klebsiella pneumoniae-associated Lemierre’s syndrome reported in the literature. DM: diabetes mellitus; F: female; I&D: incision and drainage; M: male; N: no; Y: yes. *Newly diagnosed DM within one month.

Article	Location	Age (year)/sex	Medical history	Culture-positive specimen	Complications	Surgical treatment	Anticoagulants therapy	Antibiotics therapy after known culture result	Ventilator support (due to respiratory failure)	Survival
AlAmer and AlMarzouq [7]	Saudi Arabia	46/F	N	Blood, throat culture	Lung metastasis	N	Y	piperacillin/tazobactam, vancomycin	N	Survived
Tsai et al. [8]	Taiwan	45/F	DM*, dental procedure (in previous one month)	Blood, pus	Lung metastasis	I&D	Y	Flomoxef	N	Survived
Lee et al. [9]	USA	63/F	DM, hypertension, hyperlipidemia	Blood	Lung metastasis	N	Y	Meropenem	N	Survived
Garbati et al. [10]	Saudi Arabia	63/M	DM (type I)	Pus	N	I&D	Y	Cefuroxime, clindamycin	N	Survived
Phua et al. [11]	Singapore	50/M	DM*	Blood, pus	N	I&D	Y	Cefazolin	N	Survived
Chuncharunee and Khawcharoenporn [12]	Thailand	51/F	DM, hypertension	Pus, sputum	Lung metastasis	I&D, Debridement	Y	Mpicillin-sulbactam, meropenem	Y	Died
Hwang et al. [13]	Korea	56/F	DM, hypertension, chronic otitis media	Blood, sputum	Lung metastasis, renal metastasis	N	Y	Piperacillin-tazobactam, levofloxacin	Y	Survived
Lee et al. [14]	Taiwan	56/F	N	Blood, pus	Multiple brain abscesses	I&D	N	Ceftriaxone, amikacin	N	Survived
Sabaka et al. [15]	Slovakia	19/M	N	Blood, oropharyngeal swab, pus	Spinal epidural abscess	I&D	N	Ceftriaxone, clindamycin	N	Survived
Nguyen et al. [16]	Malaysia	63/M	DM, dyslipidaemia, hypertension	Blood, pus	Lung metastasis, brain metastasis	I&D, debridement	N	Ceftriaxone, ciprofloxacin	Y	Survived
Singaporewalla et al. [17]	Singapore	68/M	DM	Blood, pus	N	I&D, Debridement	N	Amoxicillin-clavulanic acid	N	Survived
Chua et al. [18]	Malaysia	53/F	DM*	Blood	N	N	Y	Amoxicillin-clavulanic acid	N	Survived
Our case	Taiwan	43/M	DM, alcoholic liver cirrhosis	Blood, sputum	Lung metastasis	N	N	Meropenem	Y	Died

According to our review, KLS primarily affects middle-aged patients, with ages ranging from 19 to 68 years and a median age of 53, without a specific gender predominance. Unlike the classical presentation of Lemierre’s syndrome, which predominantly occurs in adolescents and young adults, particularly males [1,5,8-10]. Consistent with previous literature, the incidence of KLS is closely linked to a history of DM [7,9-13]. In our analysis, approximately 77% (n = 10) of the patients either had DM or had recently been diagnosed with it. Notably, this group includes one case involving type 1 DM [10]. Furthermore, the majority of KLS patients with DM in our dataset are from the older population.

KLS can also easily cause distant metastases, with 69% of the cases we reviewed exhibiting such metastases. Additionally, the central nervous system (CNS) (n=3) [14-16] and the kidney (n=1) [13] were also found to be affected. Among these cases, the lungs were most frequently affected (n=7) [7-9,12,13,16]. Similarly to classical Lemierre's syndrome, pulmonary metastasis remains the most common manifestation [8-10,15]. In earlier cases of Lemierre's syndrome, about 10% of the patients required mechanical ventilator support [13]. However, our statistics indicate a notably higher rate, with 31% (n=4) of KLS cases necessitating mechanical ventilator support. While the involvement of the CNS in metastatic infection is rare among patients with Lemierre's syndrome [14], our analysis indicates a higher occurrence of CNS metastasis in KLS patients, reaching 23%. In recent years, many studies have confirmed that the organs that are commonly distantly metastasized in KLS are the lungs and brain [9,12,15].

Presently, surgical drainage, in conjunction with antibiotic therapy, holds significance in the treatment approach [11,12,15]. But an important aspect of the treatment of this condition is postoperative wound management. Many of these patients may need regular debridement [17]. Currently, the recommended duration of antibiotic therapy remains in the range of 2 to 6 weeks [8,9,15-17], and the current standard for KLS is still empiric antibiotic therapy [9,12] based on the blood culture and antibiotic susceptibility analysis results. [13]. It is certain that once antibiotic therapy is delayed, the mortality rate will increase or lead to life-threatening complications [7,11]. In terms of anticoagulation therapy, the literature we reviewed indicated that due to the lack of randomized controlled trials, the current treatment recommendations for this aspect are still controversial [8-10,11,13,17,18]. Despite the intervention of surgical drainage and antibiotic treatment, the mortality rate of KLS patients in our dataset can still reach 15.4%, aligning with previous literature findings [12]. Furthermore, for patients experiencing rapid disease progression within hours, the mortality rate can surge to 75% without prompt intervention [16].

## Conclusions

Lemierre's syndrome presents as unilateral internal jugular vein septic embolism due to oropharyngeal infections, causing vein wall inflammation. It typically affects young, healthy individuals, with a higher prevalence among males. Additionally, this syndrome is associated with a high mortality rate. *K. pneumoniae* is also one of the possible pathogens that can lead to Lemierre's syndrome. However, it is a rare pathogen in Lemierre’s syndrome, with a notable incidence rate and a high mortality and ventilator support rate. The population affected by KLS differs from that of typical Lemierre's syndrome. Moreover, KLS exhibits a higher incidence in patients with a history of DM. KLS is also prone to distant metastases, especially in the lungs and brain. The cornerstone of treatment for KLS remains antibiotic therapy and surgical drainage, aligning with current practices. Extended antibiotic treatment is still recommended. Anticoagulation therapy is controversial.

Exploring the notable correlation between the population with DM and KLS is a noteworthy area for future investigation. Furthermore, the occurrence of distant metastases of KLS affecting the CNS continues to be a topic of ongoing significance. Despite the current treatment recommendations, there is a need for higher-quality research to establish the role of antibiotics, anticoagulants, and surgery in the management of KLS.

## References

[1] Lemierre A (1936). On certain septicemias due to anaerobic organisms. Lancet.

[2] Riordan T, Wilson M (2004). Lemierre's syndrome: more than a historical curiosa. Postgrad Med J.

[3] Dalen CT, Mekhail AM (2015). Lemierre syndrome: early recognition and management. CMAJ.

[4] Riordan T (2007). Human infection with Fusobacterium necrophorum (Necrobacillosis), with a focus on Lemierre's syndrome. Clin Microbiol Rev.

[5] Lee WS, Jean SS, Chen FL, Hsieh SM, Hsueh PR (2020). Lemierre's syndrome: a forgotten and re-emerging infection. J Microbiol Immunol Infect.

[6] Gore MR (2020). Lemierre syndrome: a meta-analysis. Int Arch Otorhinolaryngol.

[7] AlAmer NA, AlMarzouq WF (2023). Lemierre syndrome: a hidden complication of sore throats. Int J Emerg Med.

[8] Tsai YJ, Lin YC, Harnnd DJ, Chiang RP, Wu HM (2012). A Lemierre syndrome variant caused by Klebsiella pneumoniae. J Formos Med Assoc.

[9] Lee SE, Mushtaq A, Gitman M (2021). Lemierre's syndrome associated with hypervirulent Klebsiella pneumoniae: a case report and genomic characterization of the isolate. IDCases.

[10] Garbati MA, Ahsan AM, Hakawi AM (2012). Lemierre's syndrome due to Klebsiella pneumoniae in a 63-year-old man with diabetes: a case report. J Med Case Rep.

[11] Phua CK, Chadachan VM, Acharya R (2013). Lemierre syndrome-should we anticoagulate? A case report and review of the literature. Int J Angiol.

[12] Chuncharunee A, Khawcharoenporn T (2015). Lemierre’s syndrome caused by Klebsiella pneumoniae in a diabetic patient: a case report and review of the literature. Hawaii J Med Public Health.

[13] Hwang SY, Shin SJ, Yoon HE (2021). Lemierre's syndrome caused by Klebsiella pneumoniae: a case report. World J Nephrol.

[14] Lee WS, Wang FD, Shieh YH, Teng SO, Ou TY (2012). Lemierre syndrome complicating multiple brain abscesses caused by extended-spectrum β-lactamase-producing Klebsiella pneumoniae cured by fosfomycin and meropenem combination therapy. J Microbiol Immunol Infect.

[15] Sabaka P, Kachlíková M, Bendžala M, Káčerová H (2020). Lemierre syndrome caused by Klebsiella pneumoniae complicated by epidural abscess: case report. IDCases.

[16] Nguyen D, Yaacob Y, Hamid H, Muda S (2013). Necrotizing fasciitis on the right side of the neck with internal jugular vein thrombophlebitis and septic emboli: a case of Lemierre’s-like syndrome. Malays J Med Sci.

[17] Singaporewalla RM, Clarke MJ, Krishnan PU, Tan DE (2006). Is this a variant of Lemierre's syndrome?. Singapore Med J.

[18] Chua SH, Ong SC, Liew YH (2017). Variant of Lemierre's syndrome with internal jugular vein aneurysm. BMJ Case Rep.

